# MiR-34a Expression Has an Effect for Lower Risk of Metastasis and Associates with Expression Patterns Predicting Clinical Outcome in Breast Cancer

**DOI:** 10.1371/journal.pone.0026122

**Published:** 2011-11-10

**Authors:** Hanna Peurala, Dario Greco, Tuomas Heikkinen, Sippy Kaur, Jirina Bartkova, Maral Jamshidi, Kristiina Aittomäki, Päivi Heikkilä, Jiri Bartek, Carl Blomqvist, Ralf Bützow, Heli Nevanlinna

**Affiliations:** 1 Department of Obstetrics and Gynaecology, Helsinki University Central Hospital, Helsinki, Finland; 2 Department of Pathology, Helsinki University Central Hospital, Helsinki, Finland; 3 Department of Oncology, Helsinki University Central Hospital, Helsinki, Finland; 4 Department of Clinical Genetics, Helsinki University Central Hospital, Helsinki, Finland; 5 Institute of Cancer Biology and Centre for Genotoxic Stress Research, Danish Cancer Society, Copenhagen, Denmark; 6 Institute of Molecular and Translational Medicine, Palacky University, Olomouc, Czech Republic; Virginia Commonwealth University, United States of America

## Abstract

MiR-34a acts as a candidate tumour suppressor gene, and its expression is reduced in several cancer types. We aimed to study miR-34a expression in breast cancer and its correlation with tumour characteristics and clinical outcome, and regulatory links with other genes. We analysed miR-34a expression in 1,172 breast tumours on TMAs. 25% of the tumours showed high, 43% medium and 32% low expression of miR-34a. High miR-34a expression associated with poor prognostic factors for breast cancer: positive nodal status (*p* = 0.006), high tumour grade (*p*<0.0001), ER-negativity (*p* = 0.0002), HER2-positivity (*p* = 0.0002), high proliferation rate (*p*<0.0001), p53-positivity (*p*<0.0001), high cyclin E (*p*<0.0001) and γH2AX (*p*<0.0001). However, multivariate analysis adjusting for conventional prognostic factors indicated that high miR-34a expression in fact associated with a lower risk of recurrence or death from breast cancer (HR = 0.63, 95% CI = 0.41–0.96, *p* = 0.031). Gene expression analysis by differential miR-34a expression revealed an expression signature with an effect on both the 5-year and 10-year survival of the patients (*p*<0.001). Functional genomic analysis highlighted a novel regulatory role of the transcription factor MAZ, apart from the known control by p53, on the expression of miR-34a and a number of miR-34a targets. Our findings suggest that while miR-34a expression activation is a marker of aggressive breast tumour phenotype it exerts an independent effect for a lower risk of recurrence or death from breast cancer. We also present an expression signature of 190 genes associated with miR-34a expression. Our analysis for regulatory loops suggest that MAZ and p53 transcription factors co-operate in modulating miR-34a, as well as miR-34a targets involved in several cellular pathways. Taken together, these results suggest that the network of genes co-regulated with and targeted by miR-34a form a group of down-stream effectors that maybe of use in predicting clinical outcome, and that highlight novel regulatory mechanisms in breast cancer.

## Introduction

MicroRNAs (miRs) are short 18–24 nucleotide RNAs that work as post-transcriptional regulators by binding to sequences in the 3′ untranslated region (3′ UTR) of target mRNAs either through fully complementary or imperfect base-pairing, usually resulting in mRNA silencing [1], [2]. MiRs are estimated to regulate up to 30% of all the protein coding genes in the human genome [3]. To date, more than 9000 miRs have been identified in different species according to the miRBase release 13.0 (http://microrna.sanger.ac.uk/sequences/). Aberrant metabolism and expression of miRs have been linked to a variety of diseases, including cancer, and several miRs are thought to behave as oncogenes or tumour-suppressors as they have different expression levels in cancer as compared to normal tissues [4]. Components of the miR machinery as well as miRs themselves are involved in many cellular processes altered in cancer, such as differentiation, proliferation and apoptosis and they are demonstrated to affect cellular transformation, carcinogenesis and metastasis [5].

During the recent years, the miR-34 family has become a promising topic in cancer research [6]. This miR family consists of three members, namely miR-34a, miR-34b and miR-34c, which are encoded by two different genes: miR-34a is transcribed from its own independent locus, whereas miR-34b and miR-34c share a common primary transcript. MiR-34a resides on the chromosomal locus 1p36.23, and the loss of this region is associated with a variety of cancer types [7]. MiR-34a is highly expressed in normal tissues, like testis, lung, adrenal gland and spleen, where its physiological function is still largely unknown [8]. Its transcription is under the control of the tumour suppressor gene product p53 and it acts as a tumour suppressor inducing cell cycle arrest in G1-phase [9], [10], senescence and apoptosis [11]–[13] in osteosarcoma and breast, colon, lung and pancreatic cancer cell lines as well as in mouse tissues, such as colon, kidney, spleen and thymus. This in turn leads to reduction in the protein levels of cyclin D1 (CCND1) and cyclin-dependent kinase 6 (CDK6), which regulates the phosphorylation of retinoblastoma protein (pRb), as seen in non-small-cell lung cancer cells [14]. MiR-34a is predicted to target hundreds of mRNAs, but, to date, only a few of them have been experimentally verified, including the oncogenes MYC, CDK6, SIRT1 and MET [15], [16].

The expression of miR-34a has been observed to be reduced in many types of cancers. In epithelial ovarian cancer (EOC), the overall expression of the miR-34 family members is frequently decreased, and is associated with metastatic clinical stage and increased expression of c-MET [17]. Downregulation of miR-34a, at least partly due to mutations in p53, has been seen in the cell lines of chronic lymphocytic leukemia, as well as in pancreatic, hepatocellular and colon carcinomas [13], [18], [19]. In non-small-cell lung cancer tissue, low levels of miR-34a combined with p53 mutations were observed to correlate with a high probability of relapse [20]. In breast cancer, miR-34 levels have been found low in cell lines derived from ER/PR/HER2-negative (‘triple-negative’) tumours, which may reflect the higher incidence of p53 mutations in this subtype [21]. Furthermore, the silencing of miR-34a may also be mediated by CpG methylation of the region 100 to 500 base-pairs upstream of the miR-34a transcription start which contains a p53 binding site [22]. CpG methylation of the miR-34a promoter was also detected in 25% of breast cancer cell lines. Finally, in several cancer types including breast cancer, genomic deletions or loss of heterozygosity of the region have been described [23]. The deletion in chromosome 1p also explains the low level of miR-34a seen in neuroblastomas [24].

In this study, we focused on the expression of miR-34a in an extensive series of human breast carcinomas. We investigated miR-34a expression in breast tumours and its relationship with tumour phenotype and prognosis. Additionally, we investigated the overall transcriptional profile in tumours stratified by intensity of miR-34a expression using genome-wide DNA microarray assays. Finally, we dissected regulatory motifs that might underlie the differential expression of miR-34a and investigated the survival of the patients relative to the genes differentially expressed due to miR-34a levels.

## Methods

### Patients

A series of 884 unselected breast cancer patients was recruited at the Department of Oncology, Helsinki University Central Hospital, during the years 1997–1998 [25] and 2000 [26] (79% of all consecutive, newly diagnosed breast cancer cases during the collection periods). An additional familial breast cancer patient series (*n* = 546) was recruited at the Departments of Oncology and Clinical Genetics [27]. For the tissue microarrays (TMAs), altogether 1356 invasive breast cancer tumours were available. Detailed description of methods is included in Supporting Information (File S1).

### Ethics Statement

This study was performed with informed consent from the patients as well as permissions from the Ethics Committee E9 of the Helsinki University Central Hospital (Dnro 207/E9/07) and from the Ministry of Social Affairs and Health in Finland.

### Evaluation of immunoreactivity scores

**Figure 1:**
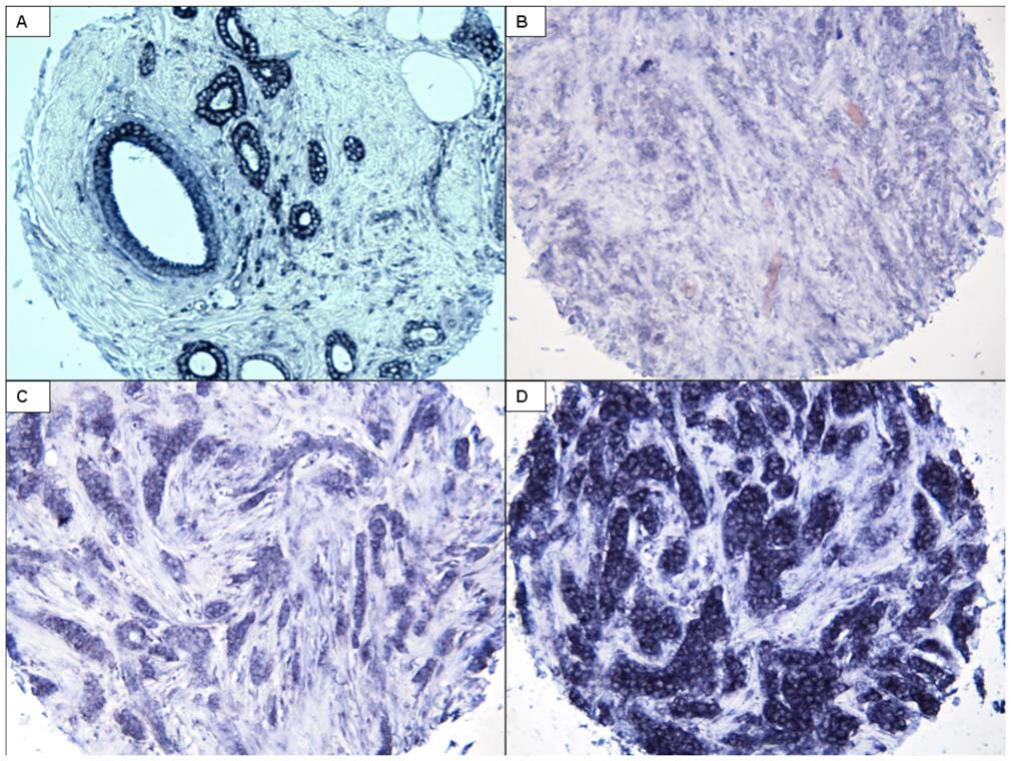
The expression of miR-34a. Benign breast epithelium (A). Category 1 (B) represents mild cytoplasmic miR-34a staining, category 2 (C) moderate staining and category 3 (D) strong staining.

Tissue microarray construction was performed as previously described [28]. The means for locked nucleic acid in situ hybridisation (LNA-ISH) for miR-34a are described in File S1. MiRs exist in the cytoplasm, as previously described, and in this study, category 1 represents weak cytoplasmic staining, category 2 moderate staining, category 3 being the highest intensity of staining (Figure 1). The positive control in our samples was a small nuclear non-coding RNA U6. The LNA probe for miR-34a used in this study has proved to be specific and functional in at least two previous studies, where the LNA-ISH results were concordant with rt-pcr and northern blot analysis [17], [29]. As a negative control we used pre-designed scrambled negative control probe (Exiqon). This probe has the same length and LNA content as the LNA detection probe and possess minimal self-annealing properties. The scrambled miR-negative control probe has been blasted in NCBI Blast for pre-miR and mature miR targets in miRBase and bears no homology to any known microRNA or mRNA-sequence.

### Relationships between miR-34a levels and clinical features

SPSS v.18.0 for MAC was used (SPSS, Inc., Chicago, IL). *P*-values for comparisons of miR-34a and tumour histopathological differences were calculated using the linear Spearman rank correlation. To account for the multiple variables tested, *p*-values<0.01 were considered significant and all *p*-values are two-sided. Kaplan-Meier survival analysis was used to estimate the effect of miR-34a on 10-year breast cancer-specific and 5-year metastasis-free or breast cancer death survival rates on different patient series. Univariate and multivariate Cox's regression analysis were used to calculate the hazard ratios for the effects of miR-34a expression on survival. ER- and PR-status were considered as categorical variables. In the multivariate analysis, T, N, M, grade, ER, PR, HER2, p53 and Ki67 were included in addition to the miR-34a result.

### Gene expression microarray analysis

Total RNA was extracted from 183 breast tumours (GEO ID GSE24450) collected at the Helsinki University Central Hospital. The samples were processed and hybridiced to Illumina Human HT-12 v3 Expression BeadChips, according to the manufacturer recommendations (http://www.illumina.com). Microarray raw data were processed by the methods included in the BioConductor facilities [30] for R v2.11 (http://cran.r-project.org). Briefly, after quality control [31] and normalization [32], the intensities of the probes mapping to the same Entrez Gene IDs [33] were averaged. A subset of 72 samples was also included in the miR-34a in situ hybridisation. In this set of tumours, moderated t-test was applied to find genes differentially expressed between the 13 samples with low miR-34a expression (in situ score 1) and the 59 samples with high miR-34a expression (in situ score 2 or 3). Genes with nominal *p*<0.01 were considered differentially expressed and further analysed. Functional annotation was performed on the differentially expressed genes using the DAVID annotation tools [34].

### Survival analysis based on the gene expression data

The miR-34a gene signature was analysed for having an effect on the clinical outcome in the larger set of 183 tumours described above (NCBI GEO accession number GSE24450) as well as in the publicly available breast cancer gene expression data set of 249 unselected primary tumours (NCBI GEO accession number GSE4922) [35]. Detailed description of these methods is contained in File S1.

### Mir-34a targets prediction

The list of differentially expressed genes was screened for potential targets of miR-34a by the integrated analysis of 9 different algorithms available at miRWalk (http://www.ma.uni-heidelberg.de/apps/zmf/mirwalk/).

### Promoter analysis

A total of 688 promoter sequences of the differentially expressed genes, including alternative promoters for the same loci, were retrieved from the Genomatix (Genomatix, Munich, Germany) and analysed for matches to the position weight matrices (PWM) for the transcription factor binding sites (TFBS) by the Genomatix MatInspector software using the default parameters [36].

## Results

The expression of miR-34a was investigated in an extensive series of breast tumours – altogether, samples from 1172 tumours were scored for miR-34a. The remaining 5.4% (*n* = 67) of the samples were not analysed due to either unpresentative or missing tissue. 25% of the tumours showed high, 43% medium and 32% low expression of miR-34a. The benign breast epithelium shows strong staining with the miR-34a probe (Figure 1).

Correlation of miR-34a expression with tumour characteristics is shown in Table 1. High miR-34a expression was associated with a non-favourable tumour phenotype of positive nodal status (*p* = 0.006), high tumour grade (*p*<0.0001), ER-negativity (*p* = 0.0002), high proliferation rate (*p*<0.0001) as well as high expression of HER2 (*p* = 0.0002), p53 (*p*<0.0001) and cyclin E (*p*<0.0001). In addition, miR-34a expression was also associated with high degree of endogenous DNA damage estimated by elevated γH2AX (*p*<0.0001), and with tumours of ductal origin (*p*<0.0001) and premenopausal status (*p* = 0.0001). High miR-34a positively correlated with high cyclin D1 among ER-positive patients (*p* = 0.0004, data not shown). However, the expression of miR-34a was not associated either with 10-year breast cancer-specific survival [cumulative survival (CS) = 78.9% vs. 84.4% for the cases with high vs. low expression of miR-34a, *p* = 0.285] or 5-year metastasis-free or breast cancer death-free survival of the patients (CS = 81.7%, vs. 84.4% *p* = 0.667). In univariate analysis performed with Cox's regression model, miR-34a showed no significant effect either on 10-year breast cancer-specific survival or 5-year metastasis-free or breast cancer death-free survival of the patients (HR = 1.16, 95% CI = 0.96–1.40, *p* = 0.12; HR = 1.09, 95% CI = 0.91–1.31, *p* = 0.372). Since miR-34a expression was strongly associated to several adverse prognostic factors for breast cancer recurrence or death, we also performed a multivariate Cox's regression analysis with these factors included in order to estimate the independent effect of the miR-34a on breast cancer survival. In contrast to the univariate analysis, the multivariate analysis showed that miR-34a expression had an independent effect on a lower risk of recurrence or death from breast cancer for the patients whose tumours had highest miR-34a expression *versus* those with lowest miR-34a expression (HR = 0.63, 95% CI = 0.41–0.96, *p* = 0.031 for the 5-year metastasis-free or breast cancer death-free survival) (Table 2). The effect on the 10-year breast cancer-specific survival was not significant although a similar tendency was seen (HR = 0.80, 95% CI = 0.50–1.25, *p* = 0.323).

**Table 1:** Association of miR-34a expression with the clinicopathological features of the tumours.

*Category*	*Total*	1	2	3	*P*	*P miR-34a*
*N (%)*	*N (%)*	*N (%)*	*N (%)*	*N (%)*		*1 vs. 2 and 3*
***Patient group***	*(n = 1172)*				0.075	0.164
Sporadic	408 (34.8)	132 (32.4)	182 (44.6)	94 (23.0)		
Large families	446 (38.1)	157 (35.2)	176 (39.5)	113 (25.3)		
Small families	318 (27.1)	85 (26.7)	143 (45.0)	90 (28.3)		
***Age***	*(n = 1172)*				**1.771^−5^**	**4.690^−4^**
<50 years	406 (34.6)	103 (25.4)	174 (42.8)	129 (31.8)		
>50 years	766 (65.4)	271 (35.4)	327 (42.7)	168 (21.9)		
***Menopause***	*(n = 811)*				**1.073^−4^**	**0.002**
Premen.	273 (33.7)	63 (23.1)	115 (42.1)	95 (34.8)		
Postmen.	538 (66.3)	181 (33.6)	230 (42.8)	127 (23.6)		
***Histology***	*(n = 1041)*				**4.543^−10^**	**1.261^−7^**
Ductal	825 (79.3)	228 (27.6)	364 (44.1)	233 (28.2)		
Lobular	216 (20.7)	100 (46.3)	91 (42.1)	25 (11.6)		
***T***	*(n = 1158)*				0.137	0.160
1	686 (59.2)	229 (33.4)	289 (42.1)	168 (24.5)		
2	399 (34.5)	118 (29.6)	174 (43.6)	107 (26.8)		
3	38 (3.3)	10 (26.3)	18 (47.4)	10 (26.3)		
4	35 (3.0)	11 (31.4)	13 (37.1)	11 (31.4)		
***N***	*(n = 1154)*				**0.006**	0.012
neg	636 (55.1)	221 (34.7)	268 (42.1)	147 (23.1)		
pos	518 (44.9)	144 (27.8)	227 (43.8)	147 (28.4)		
***M***	*(n = 1163)*				0.546	0.759
neg	1128 (97.0)	359 (31.8)	481 (42.6)	288 (25.5)		
pos	35 (3.0)	12 (34.3)	16 (45.7)	7 (20.0)		
***Grade***	*(n = 1156)*				**9.042^−21^**	**1.548^−15^**
1	281 (24.3)	127 (45.2)	114 (40.6)	40 (14.2)		
2	535 (46.3)	188 (35.1)	227 (42.4)	120 (22.4)		
3	340 (29.4)	55 (16.2)	149 (43.8)	136 (40.0)		
***ER***	*(n = 1115)*				**1.730^−4^**	**0.003**
pos	891 (79.9)	295 (33.1)	389 (43.7)	207 (23.2)		
neg	224 (20.1)	51 (22.8)	97 (43.3)	76 (33.9)		
***PR***	*(n = 1113)*				0.059	0.063
pos	748 (67.2)	246 (32.9)	321 (42.9)	181 (24.2)		
neg	365 (32.8)	100 (27.4)	164 (44.9)	101 (27.7)		
***HER2***	*(n = 1105)*				**2.368^−4^**	**1.746^−5^**
neg	960 (86.9)	322 (33.5)	402 (41.9)	236 (24.6)		
pos	145 (13.1)	23 (15.9)	77 (53.1)	45 (31.0)		
***Ki-67***	*(n = 1148)*				**1.391^−20^**	**1.132^−14^**
0	282 (24.6)	135 (47.9)	116 (41.1)	31 (11.0)		
1	492 (42.8)	152 (30.9)	217 (44.1)	123 (25.0)		
2	197 (17.2)	41 (20.8)	87 (44.2)	69 (35.0)		
3	177 (15.4)	32 (18.1)	75 (42.4)	70 (39.5)		
***p53***	*(n = 1097)*				**1.784^−7^**	**1.217^−6^**
neg	875 (79.8)	296 (33.8)	370 (42.3)	209 (23.9)		
pos	222 (20.2)	38 (17.1)	103 (46.4)	81 (36.5)		
***Cyclin E***	*(n = 1040)*				**2.827^−10^**	**2.215^−6^**
low	856 (82.3)	291 (34.0)	378 (44.2)	187 (21.8)		
high	184 (17.7)	30 (16.3)	76 (41.3)	78 (42.4)		
***γH2AX*** ***(<2% vs.*** **≥2%)**	(*n = 935*)				**9.508^−6^**	**0.001**
low	484 (51.8)	170 (35.1)	215 (44.4)	99 (20.5)		
high	451 (48.2)	114 (25.3)	191 (42.4)	146 (32.4)		

miR-34a, microRNA-34a; T, tumour size; N, nodal status; M, primary metastasis; ER, oestrogen receptor; PR, progesterone receptor; γH2AX, phosphorylated histone H2AX.

**Table 2:** Multivariate analysis of miR-34a expression with conventional prognostic factors.

10-year breast cancer-specific survival				5-year breast cancer-specific death or			
				distant metastasis-free survival			
Category	*p*-value	HR	95% CI	Category	*p*-value	HR	95% CI
M	4.279^−15^	7.36	4.47–12.13	T	3.741^−10^		
T	7.194^−8^			2 vs. 1	7.225^−6^	2.30	1.60–3.32
2 vs. 1	2.691^−4^	2.01	1.38–2.92	3 vs. 1	5.026^−9^	5.74	3.19–10.30
3 vs. 1	5.471^−6^	4.31	2.30–8.10	4 vs. 1	1.662^−6^	4.36	2.39–7.96
4 vs. 1	4.961^−7^	4.98	2.66–9.32	N	2.446^−10^	3.25	2.26–4.69
Grade	0.003			ER	0.563	1.15	0.72–1.82
2 vs. 1	0.618	1.17	0.63–2.16	PR	0.111	1.39	0.93–2.08
3 vs. 1	0.018	2.19	1.15–4.20	Grade	0.002		
N	0.000	3.09	2.11–4.54	2 vs. 1	0.013	2.30	1.19–4.42
ER	0.274	1.32	0.80–2.18	3 vs. 1	0.001	3.47	1.71–7.04
PR	0.022	1.64	1.07–2.51	Ki67	0.724		
Ki67	0.425			1 vs. 0	0.717	1.10	0.66–1.81
1 vs. 0	0.216	1.38	0.83–2.28	2 vs. 0	0.355	1.30	0.75–2.27
2 vs. 0	0.275	1.38	0.78–2.44	3 vs. 0	0.877	1.05	0.58–1.90
3 vs. 0	0.865	1.05	0.57–1.95	HER2	0.155	1.31	0.90–1.91
HER2	0.164	1.31	0.89–1.93	P53	0.476	1.14	0.79–1.66
P53	0.582	1.11	0.76–1.64	miR-34a	0.073		
miR-34a	0.573			2 vs. 1	0.065	0.70	0.48–1.02
2 vs. 1	0.773	0.94	0.64–1.40	3 vs. 1	0.031	0.63	0.41–0.96
3 vs. 1	0.323	0.80	0.50–1.25				

The table shows the results for the Cox's regression analysis of miR-34a expression with conventional prognostic factors for 10-year breast cancer-specific survival (left) and 5-year breast cancer-specific death or distant metastasis-free survival (right).

Gene expression analysis by DNA microarrays was performed on a subset of 72 samples of the 183 set, for which the miR-34a LNA-ISH score was available. We compared the samples with low miR-34a expression (LNA-ISH score 1) to the samples with high miR-34a expression (LNA-ISH score 2 or 3), in order to distinguish between loss/low miRNA expression and moderate/high expression. As many as 190 genes were retrieved as differentially expressed between these groups (Table S1). Of these, 96 and 94 genes were more highly expressed in miR-34a high and low expressing tumours, respectively. The genes more highly expressed in tumours highly expressing miR-34a represented the functional families of mitochondria (6 genes), cell cycle (7 genes), apoptosis (4 genes) and cytoskeleton (4 genes). The genes more expressed in tumours with low miR-34a expression covered the functional groups related to immune response (4 genes), cell death (6 genes), mitochondria (6 genes) and cell adhesion (4 genes) among the other functional groups (Table S2). The 190 differentially expressed genes were systematically screened in search of predicted targets of miR-34a. To this end, we used integrated prediction by nine different algorithms that screen for miRNA binding sites in the 3′-UTR of the genes. This analysis identified 43 genes with putative binding sites for miR-34a (Table S3).

**Figure 2:**
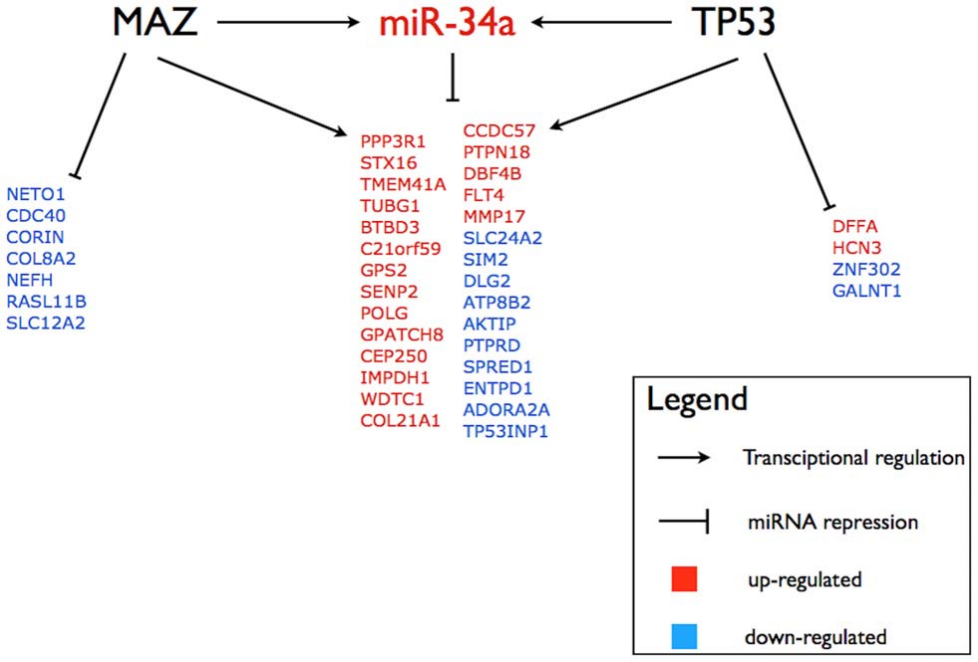
Mir-34a Feed Forward Loops (FFL). Groups of genes that are predicted to be targets of miR-34a and under the control of MAZ, p53, or both. The text color indicates the upregulated (red) and the downregulated genes (blue).

Further analysis was carried out on the promoter regions of the differentially expressed genes (Table S3). A consensus binding site for p53 was observed in 315 alternative promoters (*p* = 0.812) of 119 differentially expressed genes. Additionally, 110 differentially expressed genes were predicted to be under the transcriptional control of MAZ transcription factor (*p* = 0.053). We then looked at the genes that could form feed forward loops with miR-34a and p53, MAZ, or both. Four genes were predicted targets of miR-34a and p53; seven genes were potentially regulated by miR-34a and MAZ; twenty-nine genes were targets of miR-34a and potentially under the control of p53 and MAZ transcription factors. Genes of the cell cycle (5 genes), alternative splicing (17 genes) and apoptosis (4 genes) were potentially regulated by miR-34a as well as MAZ and p53 (Figure 2).

**Figure 3:**
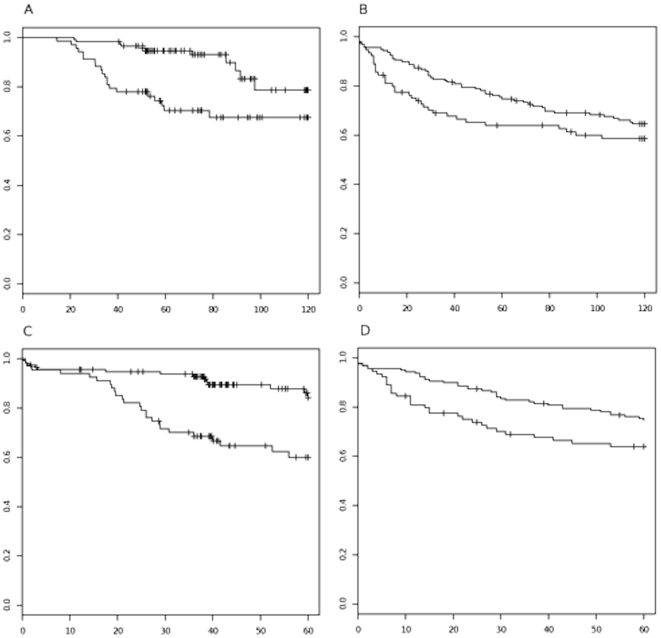
Gene signature survival analysis. Kaplan-Meier plots for 10-year breast cancer-specific survival (A) and 5-year metastasis-free or breast cancer death survival (C) in the Helsinki data set (GSE24550); 10-year breast cancer-specific survival (B) and 5-year metastasis-free or breast cancer death survival (D) in the Uppsala data set (GSE4922). For each study, the patients were split into two groups according to the expression levels of the signature genes. Subsequently, the survival rates of the two groups were compared by log-rank test.

Next, we assayed the clinical importance of the miR-34a differentially expressed genes by investigating their combinatorial effect on clinical outcome. For this purpose, we analysed the set of gene expression profiled 183 breast tumours (GEO ID GSE24450). In this dataset, the miR-34a gene signature had a significant effect on the 5-year metastasis-free or breast cancer death-free survival (*p*<0.0001) as well as on the 10-year breast cancer-specific survival (*p* = 0.0003). These results were also confirmed in a public dataset of unselected breast tumours (*n* = 249) collected at Uppsala County, Sweden, during the years 1987–1989 (GEO ID GSE4922) [35] where miR-34a gene signature showed an effect on the 5-year metastasis-free or breast cancer death-free survival (*p* = 0.038). In this Swedish dataset, the effect on the 10-year breast cancer-specific survival was not significant (*p* = 0.155) although a similar trend was seen (Figure 3).

## Discussion

To our knowledge, this is the first study investigating miR-34a expression in a large clinical series of breast tumour samples and evaluating the association of miR-34a expression with the tumour phenotype and outcome in breast cancer patients. In our dataset, low expression of miR-34a was found in about 32% and high expression in about 25% of the tumours, with the remaining tumours showing intermediate expression levels. High miR-34a expression correlated with an aggressive phenotype of hormone receptor negative tumours, p53-immunopositive, high tumour grade and high proliferation rate of the tumours. Despite association with the aggressive tumour phenotype, however, the miR-34a expression on its own was not significantly associated with either 10-year breast cancer-specific survival or 5-year metastasis-free survival in univariate analysis. Any survival effect was masked by the strong correlation of high miR-34a expression with the known prognostic factors, indicating miR-34a expression activation *per se* as a marker for an aggressive breast tumour. However, multivariate analysis, adjusting for the conventional adverse prognostic factors to evaluate the independent effect of miR-34a expression on breast cancer survival, indicated that miR-34a expression in fact was associated with a lower risk of recurrence or death from breast cancer. This finding was significant especially in the analysis of 5-year survival for distant metastasis or breast cancer specific death combined, with a similar though not statistically significant tendency on the 10-year breast cancer-specific survival. This may be due to lower statistical power in the 10-year analysis with breast cancer death as the only endpoint. In addition, the 10-year analysis could also reflect possible effects of treatment of metastatic breast cancer which might modify the patient survival in miR-34a subgroups. Overall, these results are consistent with the proposed tumour suppressor role of miR-34a and previous results on epithelial ovarian cancer showing that reduced expression of miR-34 family members is associated with metastatic clinical stage [17]. These results also suggest that miR-34a may be considered as a suppressor of metastasis, but this needs to be further evaluated in future functional studies.

The transcription factor p53 is known to bind upstream of the transcription start site of miR-34a regulating its expression [10]. Moreover, expression of miR-34a has been previously found to be reduced in 25% of breast cancer cell lines due to the methylation of its promoter [22] as well as in cell lines derived from basal-like tumours, which has been suggested to be due to the frequent p53 mutations in these tumours [21]. In our present study, 17% of tumours with positive staining for 53, suggesting p53 protein accumulation due to a mutated p53 gene, showed low miR-34a. However, as a whole, high levels of p53 protein correlated strongly with high miR-34a expression and *vice versa*, low miR-34a expression correlated with p53 negative tumours. Potential molecular basis for these findings is discussed below.

Our present study furthermore revealed that high miR-34a expression correlates with high γH2AX expression levels. The γH2AX marker reflects phosphorylation of histone H2AX by the upstream DNA damage signalling kinases ATM and ATR, and it is generally regarded as an indicator of activated response to DNA damage including replication stress, a condition shared by a wide spectrum of malignancies including breast cancer [37]–[39]. High γH2AX, as well as p53 have been implicated in DNA damage response, cellular stress and apoptosis [39], [40] and the high miR-34a expression in these tumours may reflect the response to the ongoing DNA damage and cellular stress. Interestingly, several genes among the 190 genes we found as differentially expressed in tumours subdivided by miR-34a expression are associated with functional families such as apoptosis or they encode proteins resident in the mitochondria. In addition, we identified 119 of the differentially expressed genes in tumours with different miR-34a levels to be potential targets of p53. p53 binding was not significantly enriched in the promoters of the differentially expressed genes (Table S3, enrichment *p* = 0.8), however, this is not surprising as most of the human transcription factors are expressed in most of the cells in the human body where they act as general transcription facilitators (e.g. p53) while only a small portion of them is expressed in certain conditions and devoted to more specific functions [41]. Instead, the activation of certain combinations of transcription factors lead to the expression of set of genes in specific places and conditions within the human body [42].

Previous studies have suggested that miR-34a is a target of p53 and itself acts as a tumour suppressor inducing cell cycle arrest in G1-phase [9], [10], senescence and apoptosis [11]–[13]. The unexpected correlation between high p53 protein level and enhanced miR-34a expression observed in our clinical specimens might reflect several molecular scenarios. In some tumours, positive p53 staining may reflect activation and stabilisation of a functional p53 protein in response to DNA damage and cellular stress [37], [40] and consequently p53-mediated up-regulation of miR-34a. In addition, the miR-34a itself might be under transcriptional control of also other genes than p53, and such alternative regulation may operate in the tumours where high p53 expression represents mutated dysfunctional p53. Indeed, the genome-wide mRNA profiling by miR-34a expression in our samples has highlighted a number of other differentially expressed genes whose expression might be under the control of other transcription factors (Table S3). Particularly, 110 differentially expressed genes were predicted to be under the transcriptional regulation of MAZ transcription factor (Table S3, enrichment *p* = 0.05), which is also computationally predicted to target miR-34a. Interestingly, these genes represented functional groups such as mitochondria (12 genes), cell death (10 genes) and cell cycle (7 genes) (Figure 2). MAZ gene locus maps on 16p11.2 and it has been found up-regulated in breast cancer [43]. MAZ is known to modulate PPARgamma1 (the peroxisome proliferator-activated receptor gamma 1) and down-regulation of PPARgamma1 directly, or via down-regulation of MAZ, was shown to inhibit cell growth and to induce apoptosis in MCF-7 breast cancer cells [44]. Therefore, expression of MAZ and its targets including miR-34a may play a pro-survival role in the context of breast cancer, and possibly of other tumours.

Under the assumption that co-expressed genes may also be co-regulated, overall our results suggest that the transcription of miR-34a in our breast tumour series can indeed be under the control of additional transcription factors and due to alternative regulatory circuits, and hence, further characterisation of the miR-34a regulatory region will be needed in follow-up studies. Further, taking advantage of recent data on regulatory loops involving transcription factors and miRs [45], we highlighted groups of genes forming functional feed forward loops together with miR-34a, MAZ and p53 (Figure 2). For example, high levels of miR-34a are associated with inhibition of its target AKT interacting protein (AKTIP), which is also a putative target of MAZ and p53. The AKTIP oncogene maps to chromosome 16q12.2, its product operates as part of the PI3K-AKT-pathway, and several findings link miR-34a also to this pathway [46].

On the other hand, we retrieved the metalloproteinase 17 (MMP17) to be up-regulated in the tumours with high expression of miR-34a. MMP17 was also predicted to be a target of miR-34a and potentially under of the transcriptional control of MAZ and p53. High expression levels of MMP17 have been associated with invasiveness of breast cancer, where inhibition of its expression by small interferring RNAs resulted in a non-invasive phenotype [47]. Furthermore, in tumours highly expressing miR-34a, its target TP53INP1 (p53-induced nuclear protein 1) was inhibited. TP53INP1 is a stress-induced protein involved in cell cycle arrest and apoptosis. Low levels of TP53INP1 have been observed in breast carcinoma as compared to normal breast tissue [48]. Thus, examples such as TP53INP1 would fit a potential context-dependent tumour-promoting role of miR-34a in a subset of breast tumours in vivo. In line with the emerging view of micro-RNA regulatory mechanisms, these findings suggest complex parallel or even opposing regulatory relationships both upstream and downstream of miR-34a in breast tumours that are not easily dissected in cell line models.

We further investigated the effect on the breast cancer survival and relapse of the expression pattern of the 190 genes affected by the miR-34a levels in our gene expression dataset consisting of 183 breast tumours. Additionally, an independent dataset of tumours collected at Uppsala County was also similarly tested. The aim of this analysis was to test whether the overall signature genes are able to identify groups of patients with different survival rates. Here we followed the strategies successfully utilized by Lukes et al. [49]: the patients are divided into two groups by clustering analysis based on the overall expression of the signature genes. Finally, the differential survival effect in the two patient groups is evaluated. Altogether, our results indicated that the expression signature of 190 genes is associated with the breast cancer death and relapse, especially when the effect was investigated at 5 years from the diagnosis. This suggests that the network of genes co-regulated with and targeted by miR-34a form a functional group of down-stream effectors with a prognostic effect.

In conclusion, we have shown that while miR-34a expression activation is a marker for aggressive breast cancer tumour phenotype *per se*, it excerts an independent effect for a lower risk of recurrence or death from breast cancer supporting it's proposed role as a tumour suppressor also in breast cancer. The gene expression analysis further revealed an effect of the miR-34a signature on the clinical outcome, which was also observed in an independent dataset. Our results suggest that MAZ and p53 transcription factors co-operate in modulating miR-34a, as well as the expression of several miR-34a target genes in several pathways, including PI3K-AKT, with an impact on relapse and survival of breast cancer patients. Overall, these results identify a network of genes co-regulated with and targeted by miR34a, and thereby reveal a novel aspect of breast cancer biology, with implications for prediction of clinical outcome.

## Supporting Information

File S1 **Supplementary material & methods.** A document describing the patients series and breast tumour samples, and detailed description of the methods used.
(DOC)

Table S1 **Differentially expressed genes.** The table contains the 190 genes (in rows) differentially expressed in low *versus* high miR-34a expressing tumours. The genes are ordered according to the decreasing log2 fold change. The gene names (GeneName column), the gene symbol (GeneSymbol column) and the Entrez Gene IDs (EntrezGeneID column) are provided. Additionally, the log2 fold change (logFC column), the average expression throughout the dataset (AveExpr column), the t-test values (t column) and the *p*-values (P.Value columns) are also reported.
(XLS)

Table S2 **Functional analysis of the differentially expressed genes.** The file reports the DAVID annotation tool results and it consists of 4 sheets containing the functional clustering (FClust) and the functional charts (FChart) of the upregulated genes (UP) and the downregulated genes (DN). The functional clustering tables contain the functional families (in rows) organized in groups according to the shared genes. For each cluster, the enrichment score is provided. In addition, the category, the family name (Term), the number of genes retrieved in the family (Count), the enrichment percentage (%) the enrichment *p*-value (PValue) are provided and the gene symbols (Genes) are also reported. In the functional chart tables, the functional families are ordered according to the increasing *p*-values.
(XLS)

Table S3 **Genomatix ModelInspector results and miR-34a targets.** The file consists of 5 sheets. The MatInspector table lists the transcription factor matrices (Matrix Family), the enrichment *p*-value for potential binding on the promoters of the differentially expressed genes (*P*-value), the overall number of matches (No. of matches) and the number of promoters with predicted consensus (No. of sequences). The other tables report the differentially expressed genes that are targeted by miR-34a (mir34a table), the miR-34a targets under the control of p53 (mir34a+p53), MAZ (mir34a+MAZ) or both (mir34a+MAZ+p53). The columns of these tables are as in table S1.
(XLS)
