# Supplementary material for: MiR-34a Expression Has an Effect for Lower Risk of Metastasis and Associates with Expression Patterns Predicting Clinical Outcome in Breast Cancer
Source: PLoS One. 2011 Nov 10;6(11):e26122. doi: 10.1371/journal.pone.0026122 (PMC3213093; doi:10.1371/journal.pone.0026122)
Supplement: File S1 — Supplementary material & methods. A document describing the patients series and breast tumour samples, and detailed description of the methods used. (DOC) [file pone.0026122.s001.doc]

**File S1**

**Supplementary material & methods.**

**Patients**

A series of 884 unselected breast cancer patients was recruited at the Department of Oncology, Helsinki University Central Hospital, during the years 1997–1998 [1] and 2000 [2] (79% of all consecutive, newly diagnosed breast cancer cases during the collection periods). An additional familial breast cancer patient series (*n*=546) was recruited at the Departments of Oncology and Clinical Genetics [3]. For the tissue microarrays (TMAs), altogether 1356 invasive breast cancer tumours were available. Of these, 423 were derived from patients who did not have a familial background of breast cancer (*i.e.*, sporadic) and 818 were from patients with family history of the disease. Of the cases with family history, 481 tumours were from patients with strong family history of breast cancer (at least three first- or second-degree relatives with breast or ovarian cancer, including the proband) and 337 with two affected first-degree relatives (including the proband). BRCA1- and BRCA2-mutations were screened and excluded from the familial patient series as previously described [4-6].

All cancer diagnoses were confirmed through the Finnish Cancer Registry and the non-invasive cancers were excluded. A breast cancer pathologist (PH) re-reviewed all the tumours for histology and grade. Grading was performed according to Scarff–Bloom-Richardson modified by Elston and Ellis. Information on the tumour histology, grade, size, nodal status, distant metastases, and ER- and PR-status were obtained from pathology reports [7]. For the ER- and PR-status, samples with >10% of the cancer cells stained were considered as positive. HER2-status was based on immunohistochemistry (samples with <10% of the cells stained were considered negative and >90% positive) and gene amplification with chromogenic in situ hybridisation (samples with more than six replications were considered positive and zero to five replications were considered negative) on TMA´s [8]. P53 protein expression was also investigated by immunohistochemistry [7] and samples were defined as positive for p53 when >20% of the cancer cells were stained. Ki67-status was defined with strong positive expression considered when 30%, intermediate when 20% to 29%, weak positive when 5% to 19%, and negative when <5% of the cancer cells were stained with Ki67-antibody [9]. Cyclin D1 and cyclin E analysis have also been described in Aaltonen et al. (2008). The samples with more positively stained cells than the mean value of all the samples (9.1%) were considered as high-expression tumours and the samples with less than mean value as low expression. The immunohistochemical analysis of the activated DNA damage signalling marker γH2AX was performed as described [10, 11]. Given the low background of γH2AX in normal breast tissue (0-1% of cells), values above 2% of positive cancer cells were considered as elevated.

Information on death due to breast cancer was obtained from the Finnish Cancer Registry, which collects diagnostic as well as death information on all cancer patients in Finland. The data on distant metastases was retrieved from hospital records collected with routine follow-up investigations of breast cancer patients during five years after diagnosis.

**Tissue microarray construction & locked nucleic acid in situ hybridisation (LNA-ISH) for miR-34a**

Shortly, paraffin blocks from the primary tumours were collected. Haematoxylin and eosin sections were reviewed and the most representative tumour areas were punched and placed on recipient paraffin blocks to produce TMAs consisting of four cores with diameter 0.6 mm for each tumour. Then, 3 to 4 µm thick sections were cut from array blocks and transferred to glass slides. MiR-LNA-ISH was performed on formalin-fixed paraffin-embedded breast cancer tissue microarray sections. Briefly, slides were deparaffinised with xylene, rehydrated with ethanol dilution series (100% to 25%) and treated with proteinase K (30 µg/ml; Roche) at 37°C for 10 min. After washing and postfixation in 4% paraformaldehyde, the slides were prehybridised in hybridisation buffer consisting of 50% formamide, 5xSSC, 500 ug/ml yeast tRNA, 1xDenhardt’s solution for 1 hour at a temperature of 20°C–25°C below the calculated T_m_ of the LNA probe. Slides were then hybridised in incubation chamber overnight with digoxigenin-labeled miR-LNA probe (Exiqon, Copenhagen, Denmark) diluted in hybridisation buffer. After washing (50% formamide, 2xSSC) at hybridisation temperature, slides were treated with blocking buffer (2% sheep serum, in PBST) at room temperature for 1 hour. Slides were then incubated with anti-DIG-AP Fab fragments (1:2000; Roche) and diluted in blocking buffer for overnight at 4ºC in a humid chamber. After washing the slides with PBST and AP buffer, colorimetric detection of miR-34a expression was performed using ready to use NBT/BCIP solution (1–24 hours in dark).

**Evaluation of immunoreactivity scores**

Tissue microarray construction was performed as previously described [7]. One investigator (HP) analysed the LNA-ISH scores on TMA slides and all the scoring was done under the supervision of an experienced breast cancer pathologist (RB). For the immunostainings, each core was investigated on one high-power field (25-40 X objective) and categorised in one out of three categories among the intensity of staining. MiRs exist in the cytoplasm, as previously described, and in this study, category 1 represents weak cytoplasmic staining, category 2 moderate staining, category 3 being the highest intensity of staining possible (Figure 1). The positive control in our samples was a small nuclear non-coding RNA U6. The LNA probe for miR-34a used in this study has proved to be specific and functional in at least two previous studies, where the LNA-ISH results were concordant with rt-pcr and northern blot analysis [12, 13].

**Gene expression microarray analysis**

Total RNA was extracted from 183 breast tumours (GEO ID GSE24450) collected at the Helsinki University Central Hospital. Of these, 74 were part of the series of 884 patients described above, 95 patients belonged to an additional series of sporadic patients described in [14], and the remaining 14 were familial cases. The samples were processed and hybridiced to Illumina Human HT-12 v3 Expression BeadChips, containing 24660 Entrez Gene entities, according to the manufacturer recommendations (http://www.illumina.com).

Microarray raw data were imported into R v2.11 (http://cran.r-project.org) and processed by the methods included in the BioConductor facilities [15]. Briefly, after quality control [16], the data was normalised using the quantile method [17] and the gene expression matrix was obtained by averaging the probes mapping to the same Entrez Gene IDs [18]. A subset of 72 samples was also included in the miR-34a in situ hybridisation. In this set of tumours, moderated t-test was applied to find genes differentially expressed between the 13 samples with low miR-34a expression (in situ score 1) and the 59 samples with high miR-34a expression (in situ score 2 or 3). Genes with nominal *p*<0.01 were considered differentially expressed and further analysed. Functional annotation was performed on the differentially expressed genes using the DAVID annotation tools [19].

**Survival analysis based on the gene expression data**

The miR-34a gene signature was analysed for having an effect on the clinical outcome in the larger set of 183 tumours described above (NCBI GEO accession number GSE24450) as well as in the publicly available breast cancer gene expression data set of 249 unselected primary tumours (NCBI GEO accession number GSE4922) [20]. For the GSE4922 dataset, the microarray raw data (CEL files) were retrieved from the GEO database at NCBI and preprocessed in R v2.11. Briefly, the probe sequence-based re-annotation, probe re-assignment and consequent probe set re-definition were carried out according to the last release of the Entrez Gene database [18]. Data were then background corrected, quantile normalised and summarised by the RMA algorithm [21]. For all the datasets analysed, the expression patterns for the signature genes were retrieved from the larger expression matrix and unsupervised segregative clustering (k-means) was used to assign the samples to two groups. K-means algorithm was iterated 100,000 times to ensure maximum reliability and the results were stabilised by imposing a pre-defined random number selection algorithm at the beginning of the process. The two groups of patients so formed were compared by log-rank test and the survival curves were visualised in Kaplan-Meier plots. All the analytical steps were carried out in R v2.11.

**Mir-34a targets prediction**

The list of 190 differentially expressed genes found in the gene expression microarray analysis was screened for potential targets of miR-34a by the integrated analysis of 9 different algorithms available at miRWalk (<http://www.ma.uni-heidelberg.de/apps/zmf/mirwalk/>). The genes predicted to be targets by one or more algorithms were considered for further investigation.

**Promoter analysis**

A total of 688 promoter sequences of the 190 differentially expressed genes, including alternative promoters for the same loci, were retrieved from the Genomatix (Genomatix, Munich, Germany) and analysed for matches to the position weight matrices (PWM) for the transcription factor binding sites (TFBS) by the Genomatix MatInspector software using the default parameters [22]. The *p*-values were defined as the probability to obtain an equal or greater number of sequences with a match in a randomly drawn sample of the same size as the input sequence set.

**References**

1. Syrjakoski K, Vahteristo P, Eerola H, et al. (2000) Population based study of BRCA1 and BRCA2 mutations in 1035 unselected finnish breast cancer patients. J Natl Cancer Inst 92:1529-1531.
2. Kilpivaara O, Bartkova J, Eerola H, et al. (2005) Correlation of CHEK2 protein expression and c.1100delC mutation status with tumor characteristics among unselected breast cancer patients. Int J Cancer 113:575-580.
3. Eerola H, Blomqvist C, Pukkala E, et al. (2000) Familial breast cancer in southern Finland: how prevalent are breast cancer families and can we trust the family history reported by patients? Eur. J. Cancer 36:1143-1148.
4. Vahteristo P, Eerola H, Tamminen A, et al. (2001) A probability model for predicting BRCA1 and BRCA2 mutations in breast and breast-ovarian cancer families. Br. J. Cancer 84:704-708.
5. Vehmanen P, Friedman LS, Eerola H, et al. (1997) Low proportion of BRCA1 and BRCA2 mutations in Finnish breast cancer families: evidence for additional susceptibility genes. Hum Mol Genet 6:2309-2315.
6. Eerola H, Heikkila P, Tamminen A, et al. (2005) Histopathological features of breast tumors in BRCA1, BRCA2 and mutation negative breast cancer families. Breast Cancer Res 7:93-100.
7. Tommiska J, Eerola H, Heinonen M, et al. (2005) Breast cancer patients with p53 Pro72 homozygous genotype have a poorer survival. Clin Cancer Res 11:5098-5103.
8. Tanner M, Gancberg D, Di Leo A, et al. (2000) Chromogenic in situ hybridization: a practical alternative for fluorescence in situ hybridization to detect HER-2/neu oncogene amplification in archival breast cancer samples. Am J Pathol 157:1467-1472.
9. Aaltonen K, Blomqvist C, Amine RM, et al. (2008) Familial breast cancers without mutations in BRCA1 or BRCA2 have low cyclin E and high cyclin D1 in contrast to cancers in BRCA mutation carriers. Clin Cancer Res 14:1976-1983.
10. Bartkova J, Horejsí Z, Koed K, et al. (2005) DNA damage response as a candidate anti-cancer barrier in early human tumorigenesis. Nature 434:864-870.
11. Bartkova J, Tommiska J, Oplustilova L, et al. (2008) Aberrations of the MRE11-RAD50-NBS1 DNA damage sensor complex in human breast cancer: MRE11 as a candidate familial cancer-predisposing gene. Mol Oncol 2:296-316.
12. Corney DC, Hwang CI, Matoso A, et al. (2010) Frequent Downregulation of miR-34 Family in Human Ovarian Cancers. Clin Cancer Res 16:1119-1128.
13. Wienholds E, Kloosterman WP, Miska E, et al. (2005) MicroRNA expression in zebrafish embryonic development. Science 309:310-311.
14. Fagerholm R, Hofstetter B, Tommiska J, et al. (2008) NAD(P)H:quinone oxidoreductase 1 NQO1*2 genotype (P187S) is a strong prognostic and predictive factor in breast cancer. Nat Genet 40:844-853.
15. Gentleman RC, Carey VJ, Bates DM, et al. (2004) Bioconductor: open software development for computational biology and bioinformatics. Genome Biol 5:R80.
16. Du P, Kibbe WA, Lin SM (2008) lumi: a pipeline for processing Illumina microarray. Bioinformatics 24:1547-1548.
17. Bolstad BM, Irizarry RA, Astrand M, et al. (2003) A comparison of normalization methods for high density oligonucleotide array data based on variance and bias. Bioinformatics 19:185-193.
18. Tatusova T (2010) Genomic databases and resources at the national center for biotechnology information. Methods Mol. Biol. 609:17-44.
19. Huang da W, Sherman BT, Lempicki RA (2009) Systematic and integrative analysis of large gene lists using DAVID bioinformatics resources. Nat. Protoc. 4:44-57.
20. Ivshina AV, George J, Senko O, et al. (2006) Genetic reclassification of histologic grade delineates new clinical subtypes of breast cancer. Cancer Res 66:10292-10301.
21. Irizarry RA, Hobbs B, Collin F, et al. (2003) Exploration, normalization, and summaries of high density oligonucleotide array probe level data. Biostatistics 4:249-264.
22. Cartharius K, Frech K, Grote K, et al. (2005) MatInspector and beyond: promoter analysis based on transcription factor binding sites. Bioinformatics 21:2933-2942.
